# Virtual reality environmental enrichment effects on heart rate variability in healthy volunteers

**DOI:** 10.1007/s00213-025-06870-3

**Published:** 2025-07-31

**Authors:** Giulia Benvegnù, Rudi Graffer, Federico Maria Lorusso, Sofia Ceccato, Erika Tedesco, Cristiano Chiamulera

**Affiliations:** Department of Diagnostics and Public Health, University of Verona, Verona, Italy

**Keywords:** Environmental enrichment, Heart rate variability, Virtual reality, Behavior, Psychometry, Healthy volunteers

## Abstract

**Rationale:**

Environmental enrichment (EE) is a nonpharmacological approach widely used in preclinical studies and only recently applied to humans using virtual reality (VR). Virtual EE has been shown to decrease basal cravings for smoking and palatable food; however, little is known about what processes are affected by EE. One hypothesis is that it may affect participants’ emotional state (stress- relief hypothesis).

**Objectives:**

We aimed to investigate whether physiological parameters of stress response are modified by virtual EE by assessing heart rate variability (HRV) in healthy volunteers. Second, we explored psychological measures of affective and mood states associated to virtual EE and assessed the correlation of HRV to measures of locomotion and interaction in the virtual simulation.

**Methods:**

Twenty healthy volunteers (11 men) were exposed to a virtual EE and Control Environment (CE), in counterbalancing order. HRV and participants’ behavior were measured during VR exposure. Self-report measures of mood, arousal, pleasantness and immersion were also collected before and after VR.

**Results:**

Participants showed a significant increase in time-domain HRV (RMSSD), but not in frequency-domain (HF and LF/HF ratio) measures, and self-report measures (pleasantness, activation, positive mood and perception of immersion) in EE vs. CE. Positive correlations between the score of immersion in the VR simulation and HRV indexes emerged in EE scenario only.

**Conclusions:**

The results showed an improvement in subjectively reported emotional state and an increase in parasympathetic component of HRV, suggesting that the mechanism underlying the EE effects found in this and previous work may be due to decreased stress, consistent with the “stress-relief” hypothesis.

**Supplementary Information:**

The online version contains supplementary material available at 10.1007/s00213-025-06870-3.

## Introduction

Experimental Environmental Enrichment (EE) is defined as “*a combination of complex inanimate and social stimulation*’’ (Rosenzweig et al. 1978), and consists in a spatial context that stimulate motor, sensory, and cognitive processes. Animal models of EE are characterized by a larger housing cage with a variety of objects - such as running wheels, tunnels, toys, - which stimulate the animals by providing the opportunity to perform a wide behavioral pattern (Nithianantharajah and Hannan 2006). Several studies showed that EE was able to induce changes in brain structure and functions, including improvement in learning and memory tasks, decrease of anxiety and depressive-like behaviors, reduction of drug or food -taking and -seeking (Rosenzweig and Bennett 1996; van Praag et al. 2000; Laviola et al. 2008). In animal care, EE is recommended in order to set husbandry conditions with improved welfare (Canadian Council on Animal Care 2024).

The issue is how to study the potential effects of EE and how to translate the multidimensionality of enrichment in a feasible manner in humans given the different contextual conditions compared to those in laboratory animals.

EE has been used for the rehabilitation of neuropsychological disorders (e.g., traumatic brain injuries, stroke, neurodegenerative disorders) (Alwis and Rajan 2014; Yuan et al. 2021; Cutuli et al. 2022) in order to promote brain plasticity. Moreover, EE has been found to be effective in ameliorating some of the symptoms of autism in children (Woo and Leon 2013) and is a promising behavioral treatment for substance use disorders (for a review Galaj et al. 2020; Pang et al. 2019; for a meta-analysis Wang et al. 2014;). Although the potential of EE, it is however difficult to set-up and reproduce EE conditions in the complex and variable spatial configurations of clinical intervention.

Although sensorimotor and cognitive stimulation has been used with computer games and/or task for stroke rehabilitation (e.g., Anåker et al. 2017; Janssen et al. 2012; McDonald et al. 2018), and in programs for opioid dependence (Cutter et al. 2014; Abroms et al. 2015), these computer-aided interventions do not however mimic the configurational complexity of a spatial EE conditions that would be required in an healthcare setting. Thus, the difficulty to recreate in the laboratory the complexity of such as spatial configuration urges for methodological models with both ecological validity and better control of variables and parameters (Chiamulera et al. 2017).

Virtual Reality (VR) is a technology that creates a state of immersion closer to the real situation and that allows for controlled assessment of dependent variables. In the last years, VR has been characterized as a feasible and reproducible technology for valid modelling of mental and behavioral disturbances, thus offering an experimental and therapeutic approach for several disorders (Riva et al. 2007; Bohil et al. 2011). A key feature of the VR simulations is indeed the opportunity to create contextual virtual spaces with high level of immersivity and interactivity (Chiamulera et al. 2017). For instance, VR has been used in smoking research with simulations that mimicked relapse conditions with smoking cues and context (García-Rodríguez et al. 2011; Paris et al. 2011; Pericot-Valverde et al. 2016; Benvegnù et al. 2021).

We recently investigated the effects of a virtual EE simulation on craving for virtual smoking cues and context (Benvegnù et al. 2025). Our findings showed that virtual EE might have an inhibitory effect in smokers on basal, but not on evoked, cigarette craving, similarly to effects that we have reported in a previous study for palatable food (Benvegnù et al. 2024). There is therefore the question of which processes are affected by the exposure to virtual EE under our protocol conditions. One hypothesis is that EE immersive session may affect the emotional state of the participant, whether by reducing stress. This is in accordance with the hypothesis of ‘*stress-relief*’ property of EE effect on addictive behaviors in laboratory animals (see Crofton et al. 2015; Solinas et al. 2010) which could support our previous findings on craving. As discussed in our previous paper (Benvegnù et al. 2024), we hypothesized that this “stress-relief” effect by the short virtual EE exposure, was be due to an acute and short in duration decrease of arousal.

In this pilot study, we therefore aimed to investigate whether neurophysiological parameters of stress response were modified by virtual EE by assessing heart rate variability (HRV) in healthy volunteers exposed to a virtual EE scenario similar to that described in our previous studies (Benvegnù et al. 2024, 2025). The reason to choose HRV measure is based on the established and feasible use in VR research as a physiological objective biomarker of stress response. HRV is the variation that occurs between one heart beat and another as a result of opposite sympathetic and parasympathetic control (Acharya et al. 2006). The Autonomic Nervous System (ANS) exerts control on cardiac stress response via parasympathetic and sympathetic pathways, which differently act on heart rate with the former exerting a tonic control, and the latter intervening under stress condition by increasing heart rate, concomitantly to parasympathetic withdrawal (as expressed by decreased HRV). HRV could be described under time- or frequency- domain measures. Time-domain measure is the inter-beat time, better referred as root mean square of successive differences between heart beats (RMSSD). Frequency-domain measures refer to parasympathetic control when there are high frequencies (HF) of heart rate, whereas low frequencies (LF) are under control of sympathetic pathways. The balance between stress-related sympathetic and tonic vagal parasympathetic controls is expressed as sympathovagal balance LF/HF ratio. The secondary objective was to explore psychological measures of affective and mood states associated to virtual EE (vs. non-EE control) condition. Lastly, we assessed the correlation of HRV to measures of locomotion and interaction in the virtual scenario, a behavioral assessment that has been shown to be correlated to measures of stress response in VR (Rodrigues et al. 2020).

## Materials and methods

### Participants

The experimental sample was composed of 20 healthy volunteers (age 18–65 years), native Italian language speakers (man = 11). Sample size was estimated with an a-priori analysis using G*Power 3.1 software (ANOVA: Repeated measures, within factors, α value: 0.05, power level 1-β: 0.80, effect size Cohen’s f: 0.3). The following exclusion criteria were applied: i), history of epilepsy or first-degree family members with epilepsy; ii), history of severe cardiovascular or chronic disease; iii), pregnancy; iv), presence of cardiac pacemaker or other metallic devices in the body; v), ongoing therapy with heart rate-altering drugs or with psychiatric drugs; vi), intake of psychoactive substances.

### Instrument and software

#### VR tools

We used the HTC-Vive VR device, consisting of a Head Mounted Display (HMD), two controllers for interacting with the virtual environment, and two external infrared sensors.

**Fig. 1:**
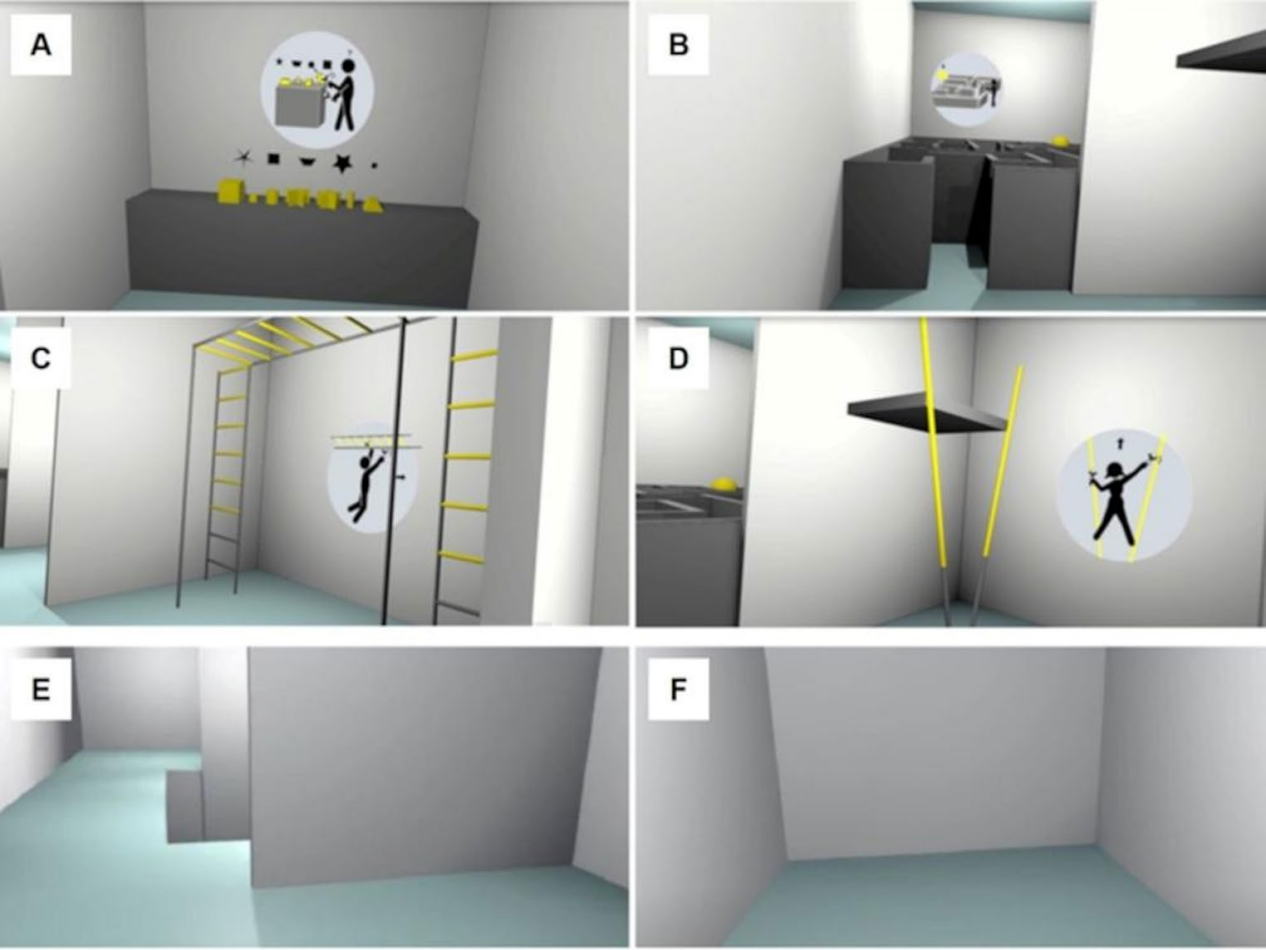
Virtual reality scenarios. EE scenario **a**), a shape matching activity, **b**), navigating a maze, **c**), a horizontal ladder climbing activity, and **d**), pole climbing; CE scenario (**e**, **f**)

All VR scenarios were modelled with Blender 2.8 software and implemented in Unity 2017.4 and have been used in previous studies (Benvegnù et al. 2024, 2025). Three scenarios were used: i), a tutorial environment to familiarize participants with VR technology, consisting of a room with a graspable object (a cube); ii), VR Enriched Environment (EE; Fig. 1); and iii), VR Control Environment (CE). The EE scenario consisted of an indoor gray walls space environment divided into four compartments, containing materials to perform two cognitive and two motor activities. The cognitive activities consisted of (a), a shape-matching activity, including mental rotation, (b), a visuomotor activity of navigating a maze. The motor activities were (c), climbing a horizontal ladder and (d), climbing poles (Fig. 1). There was copyright-free, wordless music in the headphones. The CE scenario consisted of the same indoor gray wall space environment as the EE, but devoid of the interacting objects, thus allowing no interaction.

#### Instrumentation for HRV detection

The Firstbeat Bodyguard 3 (Firstbeat, Finland), a wearable device (weight: 26 g, size: 54 mm. x 38 mm. x 7.7 mm) that records R-R interval data (sampling rate: 256 Hz) through two disposable chest electrodes placed on the heart and left fifth intercostal space, was used for HRV recording. After collection, the data were transferred to a computer using Firstbeat Bodyguard3 Exporter software, then cleaned and analyzed by using Kubios HRV standard software version 3.5.0.

#### Behavioral observation research interactive software (BORIS)

BORIS (University of Torino) was used for visual quantification of participants’ behavior during the immersion in the VR environments, using the computer screen output that corresponded to what participants viewed through HMD. We measured two parameters during the EE scenario immersion, namely Deambulation and Interaction, while in CE scenario we only measured Deambulation (no interactive objects were present). Deambulation included teleporting by using hand controller into a participant-pointed white circle on the floor of the simulation. Interaction included manipulating yellow-colored objects in the virtual environment by grasping and throwing by pulling the trigger of the hand controller in the virtual environment. Two experimenters independently assessed the behavior of the participants. The measures collected were: a), number of Interaction or Deambulation events (number of event), b), average duration of single Interaction or Deambulation events (average duration), c), percentage of session time in Interaction or Deambulation (event % time).

### Measures

#### HRV indexes

The root mean square of successive beat-to-beat interval differences (RMSSD) was used as the time-domain index, which reflects the vagal regulation of HR and is little susceptible to respiratory influences. High frequency (HF, 0.15–0.4 Hz) and ratio of low frequency (LF, 0.04–0.15 Hz) to high frequency (LF/HF), measures of vagally mediated HRV and sympathetic-vagal balance, respectively, were used as frequency-domain indices. HF absolute power (in ms^2^) and LF/HF power ratio were extracted from HRV spectrum estimates using autoregressive (AR) modelling based spectrum estimation method.

#### Mood

The positive and negative mood associated with each virtual environment was measured after each scenario using ad hoc 10-point scales (0 = Strongly disagree, 9 = Strongly agree) built on the basis of the literature (Benvegnù et al. 2021, 2025) with items such as “I am happy, cheerful or satisfied”.

#### Pleasantness and arousal

Pleasantness and arousal elicited by the virtual scenarios were measured using ad hoc 10-point scales (0 = Not at all, 9 = Extremely) based on the literature (Vecchiato et al. 2015; Benvegnù et al. 2021, 2025) and characterized by items such as “Can you rate how much you liked this environment?“.

#### Immersion in VR environments

The degree of immersion elicited by each virtual environment was measured after each scenario using an ad hoc 10-point scale built on the basis of the literature (Vecchiato et al. 2015; Benvegnù et al. 2021, 2025), consisting of the item “To what extent did you feel immersed in the environment you just perceived?”.

#### Side effects associated with VR environments

The presence of symptoms associated with cybersickness was assessed using the Simulator Sickness Questionnaire (SSQ, Kennedy et al. 1993). The SSQ consists of 16 items (a list of symptoms such as “Fatigue,” “Headache” and “blurred vision.“), 4-point response scale (from “None” to “Severe”), and provides a total score and 3 subscales (Nausea, Oculomotor Disturbance, and Disorientation). Cronbach’s Alpha is 0.84.

### Procedure

**Fig. 2:**
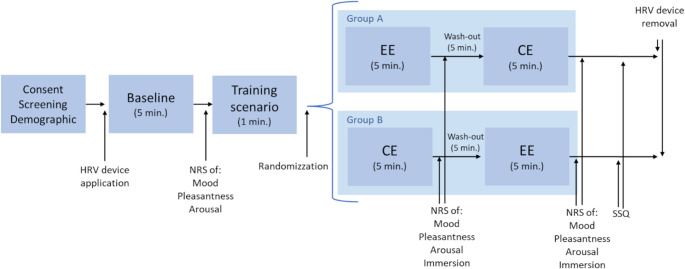
Schematic diagram of the protocol. Abbreviations: CE = Control Environment; EE = Environmental Enrichment; HRV = Heart Rate Variability; NRS = Numeric Rating Scale; SSQ = Simulator Sickness Questionnaire

Volunteers were asked not to take nicotine or caffeine during the hour prior to the study. All participants underwent an initial screening during which they received the necessary information and signed informed consent. HRV instrumentation was then applied to each participant. After 5 min of baseline recording, the participant was instructed on how to move around in a VR environment and how to use the controllers. To familiarize with VR, each participant was immersed in a training scenario for the duration of 1 min. Participants were then randomly assigned to either group A or group B and then immersed in EE and CE, for 5 min each, according to the order provided by their group. During the VR immersion, video recording was made of what the participant was seeing. Participants were not asked to perform specific tasks during immersion in virtual environments but were left free to move and grasp objects they found within the specific immersion condition. At the end of the baseline and each scenario, they filled out Numeric Rating Scales (NRS) to measure mood state, pleasantness, arousal and perceived immersion state. At the end of the experimental session, each subject also filled out the Simulator Sickness Questionnaire (SSQ) to check for any side effects associated with VR (Fig. 2).

### Data analysis

HRV data were continuously collected during immersion in virtual environments. Then, the RMSSD, HF and LF/HF were extracted at the baseline, and during the 5-minute time window spent by participants in the virtual EE and CE. Detection of outliers and artifacts was first performed by visual inspection, and when necessary, a correction algorithm was used in which artifacts were detected from a time series consisting of differences between successive RR intervals. For each participant on whom such correction was made, the percentage of corrected beats never exceeded 5% of the signal. In total, *n* = 77 beats were corrected. Data from one participant were excluded in the analyses of physiological indices because of insufficient signal quality.

HRV indexes were analyzed by One-Way repeated measures ANOVA (three levels: baseline, EE, CE) with Geisser-Greenhouse correction or nonparametric alternatives (Friedman test) in case of non-normal data (Shapiro-Wilk test). In case of significance, multiple within-group comparisons (with Holm-Šídák correction for ANOVA and corrected Dunn’s multiple comparisons test for Friedman test) were performed, with a focus on the key comparison between EE and CE. Confidence intervals of the post-hoc tests without the Holm-Šídák correction will be reported, as this correction adjusts the p-values of the individual comparisons to control the family -wise error rate (FWER) and does not allow for confidence intervals. The same analysis was conducted for mood state, pleasantness, and arousal. After verifying the non-normality of the data (Shapiro-Wilk test), the sense of presence and the BORIS measures of Deambulation were analyzed with Wilcoxon Signed Rank test, while SSQ was analyzed with one sample Wilcoxon Signed Rank test (comparison with scale median).

To test for associations between the different levels of response collected, Pearson or Spearman correlations (in case of non-normal data) were performed between self-reports and HRV indices and between self-reports and BORIS measures.

All statistical analyses were performed with GraphPad Prism version 9.1.0.

## Results

### HRV indexes

**Fig. 3:**
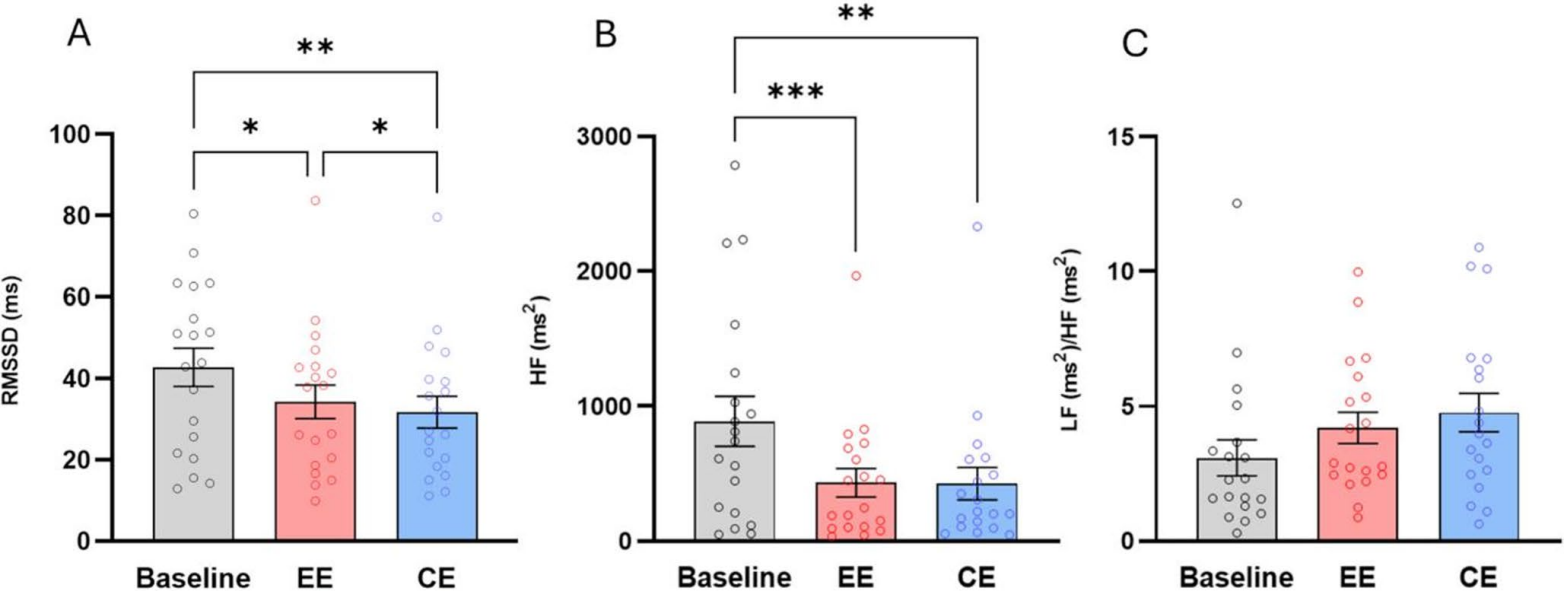
Mean values and Standard Error of the Mean (± S.E.M.) of (**a**) RMSSD, (**b**) HF and (**c**) LF/HF measured during baseline, enriched (EE) and control environment (CE). * (*p* < 0.05), ** (*p* < 0.01), *** (*p* < 0.001) Holm-Šídák post-hoc test. Abbreviations: RMSSD = root mean square of successive beat-to-beat interval differences, HF = High frequency; LF = Low frequency

The One-Way ANOVA performed on the RMSSD was statistically significant [F (1.17, 21.17) = 11.45, *p* = 0.001]. Post-hoc comparisons (Fig. 3A) showed significantly higher values in the baseline compared with the EE scenario [t (18) = 2.860, *p* = 0.02, 95% CI (2.257, 14.75), Cohen’s d = 0.64] and the CE scenario [t (18) = 3.983, *p* = 0.002, 95% CI (5.217, 16.86), Cohen’s d = 0.91]. The key comparison between CE and EE was also significant. In fact, compared with the CE scenario, the EE scenario showed a significantly higher RMSSD [t (18) = 2.539, *p* = 0.02, 95% CI (0.437, 4.635), Cohen’s d = 0.57].

Both Friedman’s test performed on HF [χ2(3) = 18.00, *p* = 0.0001] and LF/HF [χ2(3) = 6.42, *p* = 0.040] were significant. Post-hoc comparisons (Fig. 3B and C) showed only higher HF at baseline compared with CE (z = 3.893, *p* = 0.002, *r* = 0.89) and EE (z = 3.407, *p* < 0.001, *r* = 0.78), while no significant differences emerged for the key comparison between CE and EE (z = 0.448, *p* > 0.9, *r* = 0.11). As for LF/HF, neither the comparison between baseline and CE (z = 2.271, *p* = 0.06, *r* = 0.52) and baseline and EE (z = 2.109, *p* = 0.1, *r* = 0.48), nor the key comparison between CE and EE (z = 0.162, *p* > 0.9, *r* = 0.03) were significant.

**Fig. 4:**
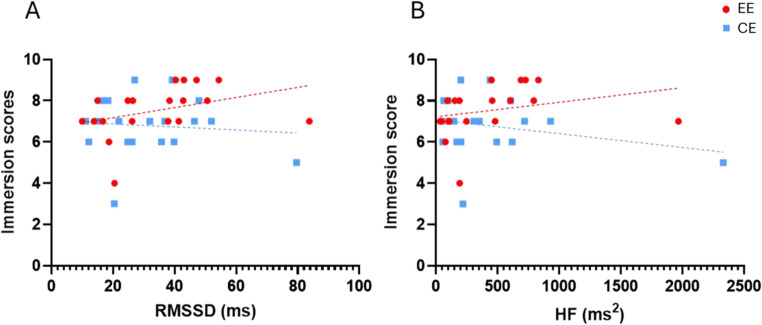
Correlational analysis, **a**), between RMSSD (X-axis) and immersion scores (Y-axis) and, **b**), between HF (X-axis) and immersion scores (Y-axis) measured in the enriched environment (red dots) and in the control environment (blue squares). Abbreviations: HF = High frequency; RMSSD = root mean square of successive beat-to-beat interval differences; EE = Enriched Environment; CE = Control Environment

Pair-wise correlational analysis (Spearman’s r) between HRV indices and self-reports revealed significant correlations only between immersion scores and RMSSD [*r* = 0.52, *p* = 0.020, 95% CI (0.08, 0.79)] and HF [*r* = 0.50, *p* = 0.027, 95% CI (0.05, 0.78)] measured in the EE (Fig. 4). No significant correlations were found in the CE. Full results are given in Table S1 in the supplementary materials.

### Questionnaires

#### Positive and negative mood

**Fig. 5:**
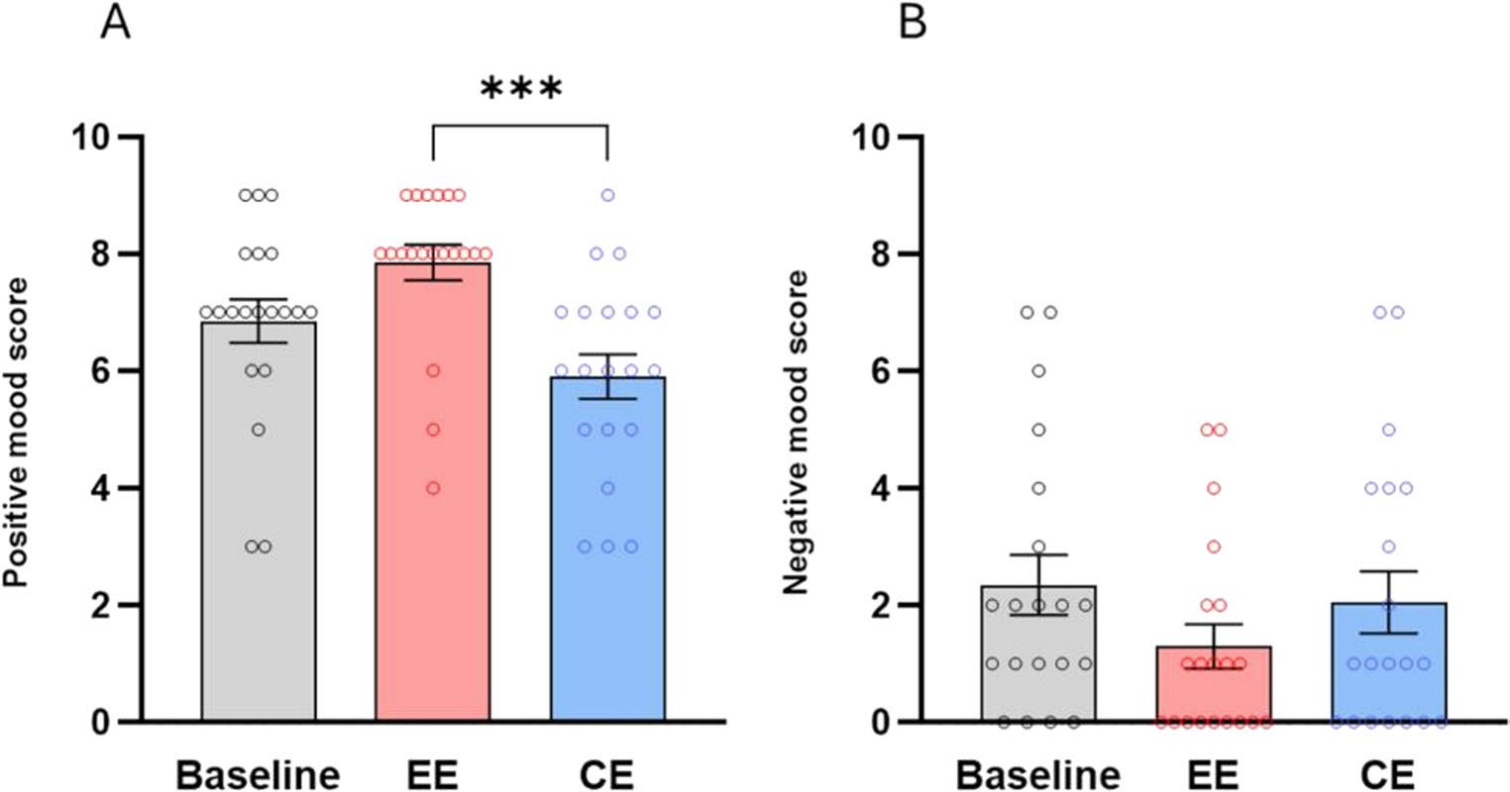
Mean scores and Standard Error of the Mean (± S.E.M.) of, **a**) positive mood and, **b**), negative mood measured after baseline, enriched (EE) and control environment (CE). *** (*p* < 0.001), Dunn’s multiple comparisons test

Friedman’s test performed on positive mood was significant [χ2(3) = 19.94, *p* < 0.001]. Post-hoc tests revealed a significantly higher score in the EE scenario than in the CE (z = 3.953, *p* < 0.001, *r* = 0.88) (Fig. 5). Although Friedman’s test related to negative mood was also significant [χ2(3) = 7.62, *p* = 0.022], post-hoc comparisons did not show significance.

#### Pleasantness and arousal

**Fig. 6:**
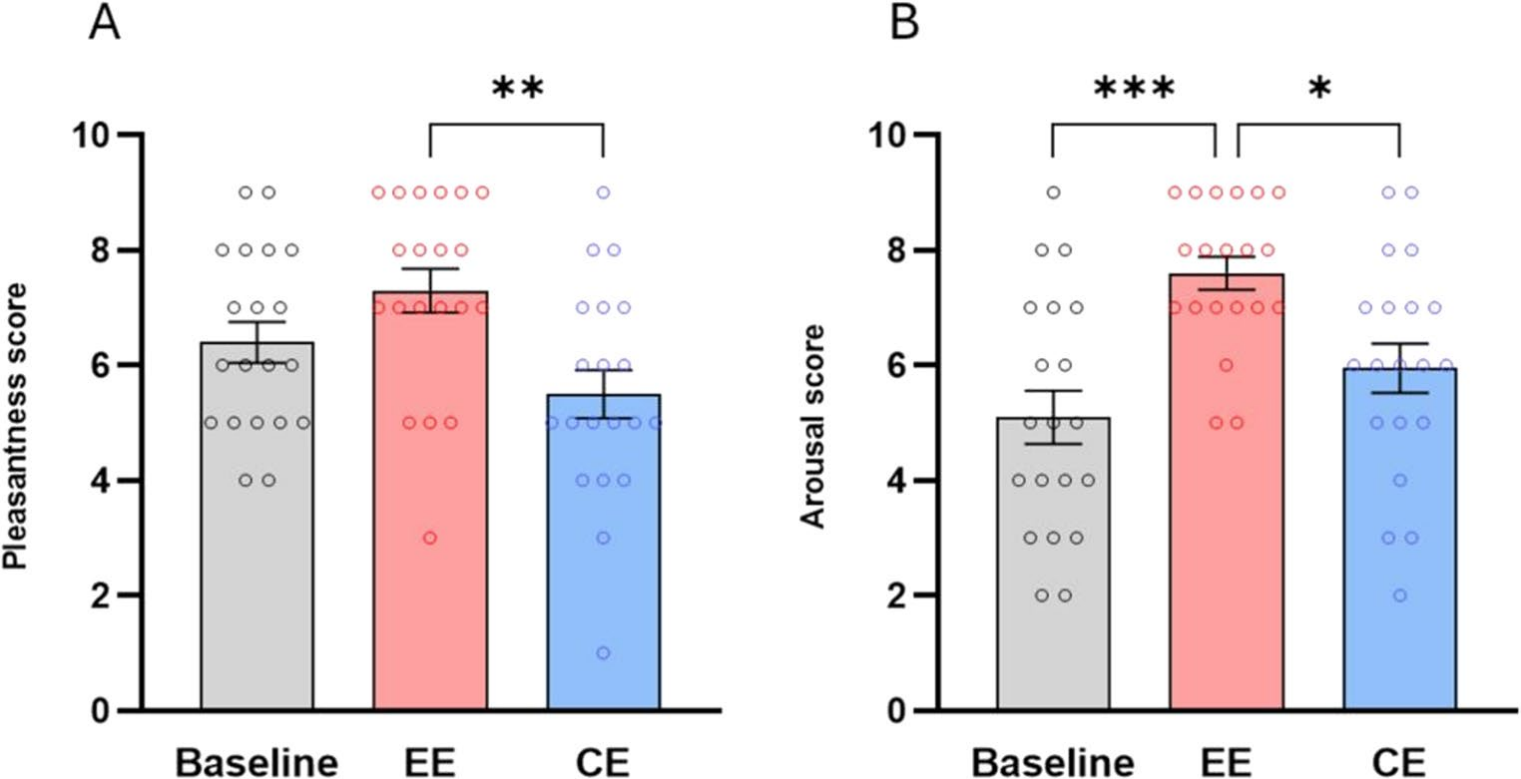
Mean scores and Standard Error of the Mean (± S.E.M.) of: (**a**) pleasantness and (**b**) arousal measured after baseline, enriched (EE) and control environment (CE). * (*p* < 0.05), ** (*p* < 0.01) and *** (*p* < 0.001), Dunn’s multiple comparisons test

Friedman’s tests performed on pleasantness [χ2(3) = 16.41, *p* < 0.001] and arousal [χ2(3) = 20.53, *p* < 0.001] scores were significant. Post-hoc tests showed significantly greater pleasantness and arousal in the EE scenario than in the CE (pleasantness: z = 3.558, *p* = 0.001, *r* = 0.79; arousal: z = 2.925, *p* = 0.01, *r* = 0.65) (Fig. 6). In addition, EE-related arousal scores were also significantly higher than baseline activation (z = 4.190, *p* < 0.001, *r* = 0.93).

#### Immersion and side effects

**Fig. 7:**
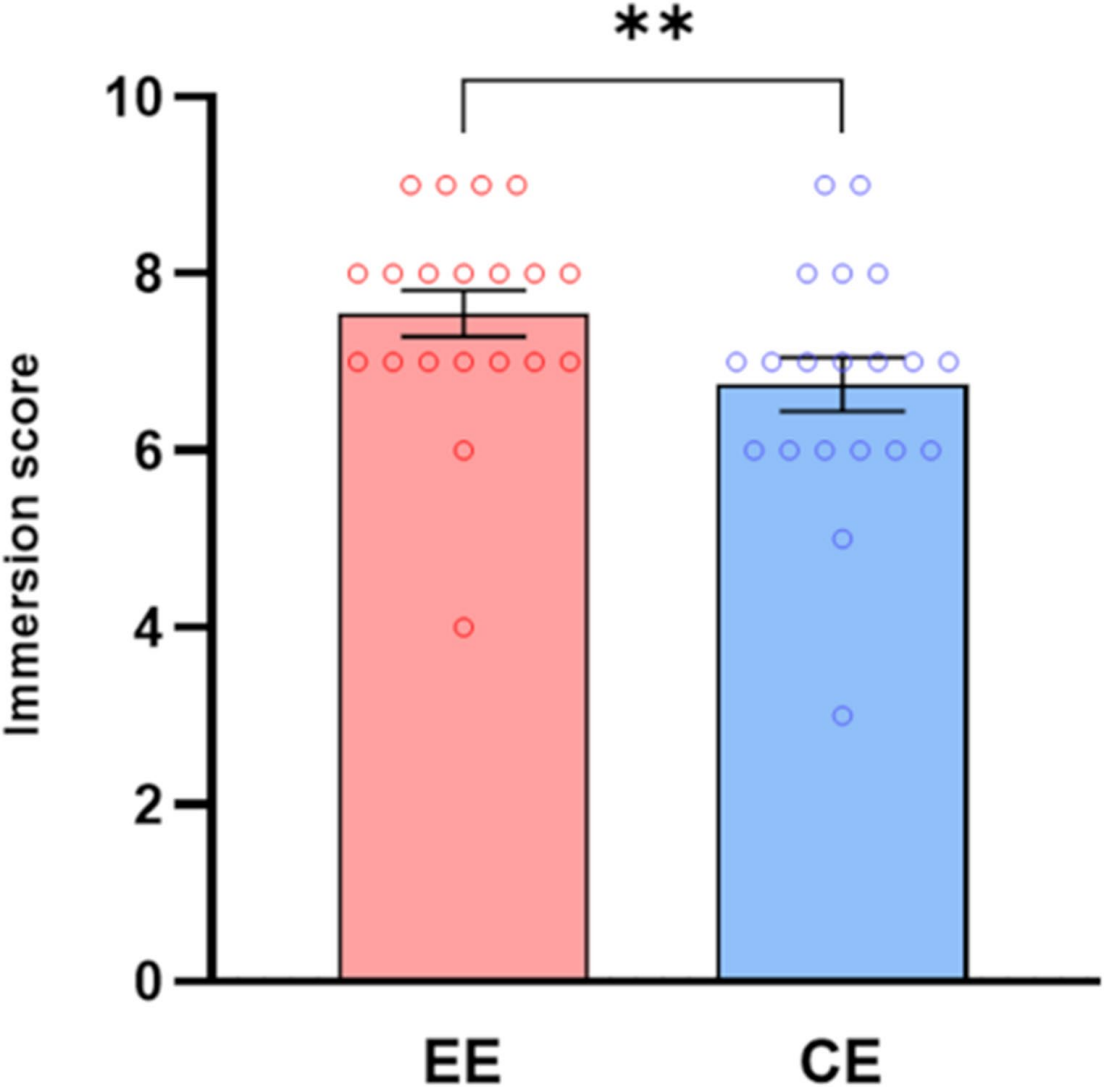
Mean scores and Standard Error of the Mean (± S.E.M.) of the immersion scores measured after enriched (EE) and control environment (CE). ** (*p* < 0.01), *** (*p* < 0.001), Wilcoxon signed rank test

The Wilcoxon signed rank test performed on the immersion scores was significant (z = 2.631, *p* = 0.008, *r* = 0.67) (Fig. 7). In contrast, no significantly above-average side effects emerged from the one sample Wilcoxon signed rank test performed on the SSQ (*p* = 0.2).

### Activity in virtual environments

**Fig. 8:**
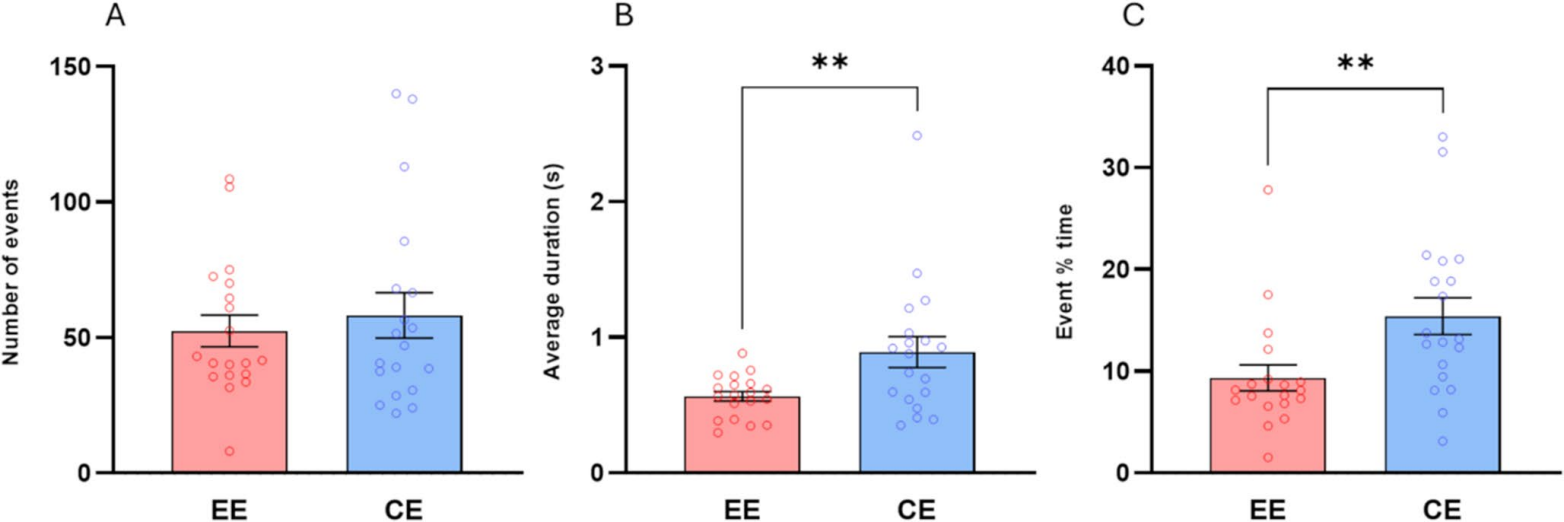
Mean scores and Standard Error of the Mean (± S.E.M.) of, **a**), the number of events, **b**), the average duration and, **c**), the percentage of session time spent walking (event % time) measured in enriched (EE) and control environment (CE). ** (*p* < 0.01), Wilcoxon signed rank test

The Wilcoxon signed rank tests performed on the different walking indices revealed that although the number of events did not differ significantly between EE and CE (*p* = 0.4), the average duration (z = 2.984, *p* = 0.003, *r* = 0.68) and the percentage of session time spent walking (z = 2.738, *p* = 0.006, *r* = 0.62) were significantly higher in the CE scenario than in the EE scenario (Fig. 8). Additional analyses of the activity in the virtual environments divided by groups can be found in the supplementary materials.

Pair-wise correlational analysis (Spearman’s r) between the measures of Deambulation and self-reports revealed significant correlations between pleasantness scores and number of events [*r* = 0.51, *p* = 0.024, 95% CI (0.066 to 0.79)] measured in the EE. No other significant correlations with other self-report measures emerged in either the enriched or control environment.

**Fig. 9:**
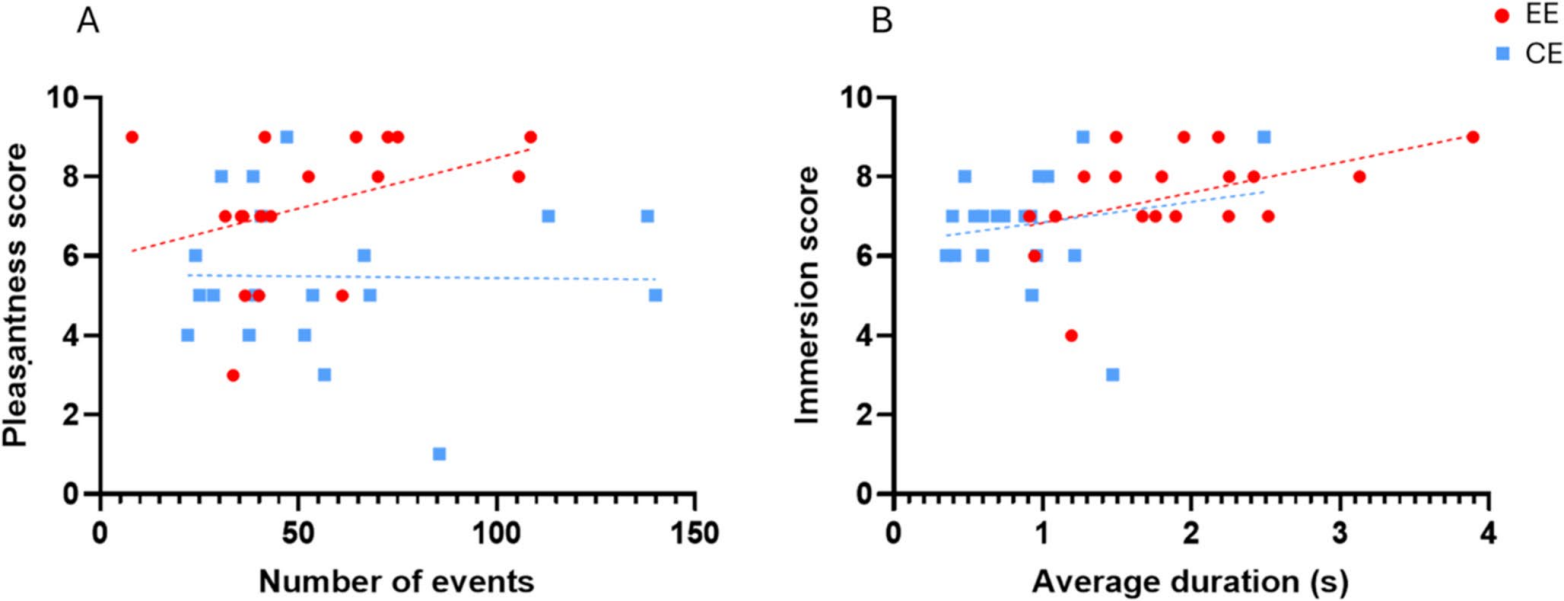
Correlational analysis, **a**) between number of deambulation events (X-axis) and pleasantness scores (Y-axis) and, **b**), between average interaction duration (X-axis) and immersion scores (Y-axis) measured in the enriched environment (EE, red dots) and in the control environment (CE, blue squares)

Pair-wise Spearman correlation between the measures of Interaction in EE and self-reports revealed a positive significant correlation between average duration and immersion [*r* = 0.46, *p* = 0.044, 95% CI (0.0002, 0.76)] (Fig. 9). Full results are given in Table S2 in the supplementary materials.

## Discussion

In the present pilot study, we investigated in healthy participants which processes are affected by immersion in a virtual EE by testing neurophysiological parameters of stress response (i.e., HRV), subjective psychological measures of affective states (mood, pleasantness, arousal, and degree of perceived immersion), and behavioral activity. We found increased heart rate variability (RMSSD), positive mood, arousal, pleasantness and immersion in EE compared to CE. Furthermore, in EE only group we observed positive correlations between physiological measures of parasympathetic activation (i.e., RMSSD and HF) and degree of perceived immersion, as well as between behavioral measures and perceived pleasantness and immersion.

Recently, HRV has been widely used as a physiological measure of stress in virtual reality immersions, in particular as a correlate of relaxation or biofeedback responses (Rockstroh et al. 2019; Dammen et al. 2022; Spano et al. 2023; Pratviel et al. 2024). For this purpose, the use of natural VR scenarios showed increased HRV indices reflecting augmented parasympathetic activity - such as HF (Aganov et al. 2020) and RMSSD (Yin et al. 2020; Kumpulainen et al. 2024) - or decreased HRV indices associated with sympathetic activation - such as LF and LF/HF ratio (Kim et al. 2021). Since the balance between stress-related sympathetic and tonic vagal parasympathetic controls is expressed as sympathovagal balance LF/HF ratio, a decrease of this ratio is expression of parasympathetic tonic reduction with ‘substitution’ by sympathetic HR control.

In our study we observed an increase of RMSSD in the EE compared to the control CE condition, expression of a greater vagal control, which was also correlated to positive affective and mood measures. It is however important to highlight that compared to baseline, both the VR conditions induced a decrease of RMSSD and HF, suggesting that the exposure to VR immersion induced a reduction of vagal control, and possibly a sympathetic substitution as shown by LF trend to increase. A partial interpretation of our data indicates that the significant difference in RMSSD between the two experimental groups suggest that the virtual EE condition is ‘less stressful’ than the CE compared to baseline, conclusion that is not however supported by significant difference either in HF or LF/HF ratio indices between the two groups.

Our findings are in contrast to studies performed under VR time-pressure tasks, but not under simple immersion in a non-interactive virtual environment (Malińska et al. 2015). Similarly, high-difficulty tasks performed in VR induced decreased HF compared to simple exercises (Rodrigues et al. 2021). Thus, the type of VR engagement and tasks difficulty appears to be virtual contextual factors that could differentially affect HRV changes. It should be noted that participants were free to behave in our virtual environment, and there was no obligation or time pressure in carrying out the tasks. It can therefore be hypothesized that the participants were stimulated to interact by the ‘enriched’ features of the environment, as can also be seen from the immersion scores and correlations of these with the HRV indices, but without being stressed. In line with this hypothesis, the study of Liszio and Masuch (2019) showed how the interaction with the elements of a virtual environment produces a greater state of immersion and decreases stress compared to passive exposure (Liszio and Masuch 2019). The type of VR immersion task, and the subjective affective state of participants in our study may explain the lack of significant effects on frequency-domain measures HF and LF. It should be considered however that literature evidence suggest that on short lasting measurement periods, time-domain RMSSD is more reliable than frequency-domain measures (Shaffer and Ginsberg 2017). Indeed, there is an open debate in literature on the utility of the LF and LF/HF ratio measures, more reliable on long-term recordings, as index of HRV in brief sessions (Billman 2013). It is also important to highlight that our aim was to test the acute, ad-hoc, effect of EE on a rapid physiological and psychological response. Our research question (see also our previous works by Benvegnù et al. 2024, 2025) was whether an ad-hoc enriching experience might – hypothetically through a stress- relief effect – help to reduce basal and evoked cigarette or palatable food craving.

Positive mood and pleasantness ratings in the EE group suggest a beneficial impact on participants’ emotional state and a greater engagement with tasks compared to control. Additionally, as more participants engage in task completion and interact with the EE, immersion levels rise as reflected in the time devoted to each activity.

Our results are in agreement with our previous studies, where brief exposure to a virtual EE led to reductions in the POMS fatigue score (indicating lower stress and exhaustion levels) and in baseline craving for palatable foods in healthy volunteers (Benvegnù et al. 2024), as well as baseline craving in smoking volunteers (Benvegnù et al. 2025), suggesting a stress-relief effect. It was not possible in these previous studies to detect the specific physiological response due to the EE exposure only without the concomitant, confounding, effects of factors such as type of participants, cue reactivity states, etc. Therefore, the scope of the present study was to design a simple protocol for assessing the specific HRV response to the single EE exposure, in healthy (i.e., without confounding factors) participants. That said, and based on the findings of our previous studies on cue reactivity, we could speculate that in patients virtual EE may improve affective, and mood subjective state (as shown by psychological measurements) partially correlated to the physiological stress-related measure of HRV described in the present study.

In an elegant paper by Rodrigues et al. (2020), the assessment of HRV during the exposure to a virtual novel environment was shown to be a predictive measure of stress response in a threatening virtual scenario, and be proposed as a non-invasive biomarkers of disease. As described by the Authors, the HRV measure was more accurate than psychometric questionnaires. In the same study, individual changes on HRV correlated with the locomotor activity of the participants in the experimental room. In spite of the different virtual exposure modality (locomotion measured by video recording of activity in the virtual scenario vs. activity by sensors in the real experimental room, respectively in our and Rodrigues et al. paper), our results suggest an agreement on the feasibility and validity of physiological vs. behavioral correlational analysis approach. Our measurement of behavioral activity during the virtual session may not be considered a predictive marker since the assessment was concomitant with HRV recording (differently from Rodrigues et al. 2020). In our experiment, however, the early, faster, HRV physiological response has anyway temporally anticipated the behavioral activity. This correlation may therefore suggest an underlying mechanistic feature of EE exposure, that may probably be identified in stress-regulation processes.

A main limitation of our study is the traditional visual measurement of behavior. A machine learning approach of a larger set of data (see Rodrigues et al. 2020) would have given a more sensitive readout of the activity of the subjects in the different VR scenario conditions, and possibly a different pattern of significant correlations. With the same approach, and with a larger sample size, more insight it would have been obtained on interindividual variability. A second limitation is the small sample size, an issue that we tried to mitigate by adopting a within-subject design. The small sample size might also be an alternative reason for the lack of significant differences of HF and LF between groups. In addition, the small sample allowed only large effects to be detected by correlational analysis. It is possible that other correlations would have emerged as significant with a more adequate sample size. Finally, using only cardiac measures is a limitation that future studies can overcome by adopting other physiological indices of stress besides HRV (e.g., salivary cortisol, skin conductance). Stress responses involve the activation of the Hypothalamic Pituitary Adrenal (HPA) axis and Autonomic Nervous System, commonly assessed as cortisol level and galvanic skin response (GSR) or HRV respectively.

The different temporal scale of change is the criterium for choosing among these different assessments, with HPA cortisol activation requiring tens of minutes and GSR/HRV seconds to minutes. Cortisol response may therefore be a biomarker of both acute and sustained physiological response to stress (Aguilera 1994). GSR is a parasympathetic and fast response biomarker like HRV, but it has also been associated to orienting responses and thus on attention (Boucsein 2012).

In conclusion, in the present pilot study, we tested whether a VR-enriched environment could influence the physiological stress response, as measured by HRV, in healthy volunteers. We found increased RMSSD, as well as increased pleasantness, activation, positive mood and perception of immersion compared with the control environment, suggesting that the mechanism underlying the environmental enrichment effects found in this and previous works may be due to decreased stress, consistent with the “stress-relief” hypothesis.

## Supplementary Information

Below is the link to the electronic supplementary material.

Supplementary Material 1

## Data Availability

Data available on request to authors.

## References

[CR1] Abroms LC, Leavitt LE, Van Alstyne JM et al (2015) A motion videogame for opioid relapse prevention. Games Health J 4:494–501. 10.1089/g4h.2014.0100 26213838 10.1089/g4h.2014.0100 PMC4675183

[CR2] Acharya UR, Joseph KP, Kannathal N et al (2006) Heart rate variability: A review. Med Biol Eng Comput 44:1031–1051. 10.1007/s11517-006-0119-0 17111118 10.1007/s11517-006-0119-0

[CR3] Aganov S, Nayshtetik E, Nagibin V, Lebed Y (2020) Pure purr virtual reality technology: measuring heart rate variability and anxiety levels in healthy volunteers affected by moderate stress. Arch Med Sci 18:336. 10.5114/aoms.2020.93239 35316901 10.5114/aoms.2020.93239 PMC8924843

[CR4] Aguilera G (1994) Regulation of pituitary ACTH secretion during chronic stress. Front Neuroendocrinol 15:321–350. 10.1006/frne.1994.1013 7895891 10.1006/frne.1994.1013

[CR5] Alwis DS, Rajan R (2014) Environmental enrichment and the sensory brain: the role of enrichment in remediating brain injury. Front Syst Neurosci 8:1–20. 10.3389/fnsys.2014.00156 25228861 10.3389/fnsys.2014.00156 PMC4151031

[CR6] Anåker A, Von Koch L, Sjöstrand C et al (2017) A comparative study of patients’ activities and interactions in a stroke unit before and after reconstruction - The significance of the built environment. PLoS ONE 12:1–12. 10.1371/journal.pone.0177477 10.1371/journal.pone.0177477 PMC5519004 28727727

[CR7] Benvegnù G, Tommasi F, Ferraro S et al (2021) Smokers context reactivity in virtual domestic environments. Eur Addict Res 27:439–446. 10.1159/000515301 33940577 10.1159/000515301

[CR8] Benvegnù G, Piva A, Cadorin C et al (2024) The effects of virtual reality environmental enrichments on craving to food in healthy volunteers. Psychopharmacology (Berl) 241:49–60 37697163 10.1007/s00213-023-06462-z PMC10774167

[CR9] Benvegnù G, Perotti S, Vegher A, Chiamulera C (2025) Virtual reality environmental enrichment effects on craving for cigarette in smokers. Games Health J 14:21–28. 10.1089/g4h.2023.0188 38985569 10.1089/g4h.2023.0188

[CR10] Billman GE (2013) The LF/HF ratio does not accurately measure cardiac sympatho-vagal balance. Front Physiol 4 FEB:1–5. 10.3389/fphys.2013.00026 23431279 10.3389/fphys.2013.00026 PMC3576706

[CR11] Bohil CJ, Alicea B, Biocca FA (2011) Virtual reality in neuroscience research and therapy. Nat Rev Neurosci 12:752–762. 10.1038/nrn3122 22048061 10.1038/nrn3122

[CR12] Boucsein W (2012) Electrodermal Activity, 2nd edn. Springer, New York

[CR13] Canadian Council on Animal Care (2024) Environmental Enrichment. https://ccac.ca/en/training/modules/animals-housed-in-vivaria-stream/environmental-enrichment.html. Accessed 16 Oct 2024

[CR14] Chiamulera C, Ferrandi E, Benvegnù G et al (2017) Virtual reality for neuroarchitecture: cue reactivity in built spaces. Front Psychol 8:1–5. 10.3389/fpsyg.2017.00185 28243216 10.3389/fpsyg.2017.00185 PMC5303754

[CR15] Crofton EJ, Zhang Y, Green TA (2015) Inoculation stress hypothesis of environmental enrichment. Neurosci Biobehav Rev 49:19–31. 10.1016/j.neubiorev.2014.11.017 25449533 10.1016/j.neubiorev.2014.11.017 PMC4305384

[CR16] Cutter CJ, Schottenfeld RS, Moore BA et al (2014) A pilot trial of a videogame-based exercise program for methadone maintained patients. J Subst Abuse Treat 47:299–305. 10.1016/j.jsat.2014.05.007 25012555 10.1016/j.jsat.2014.05.007 PMC4487635

[CR17] Cutuli D, Landolfo E, Petrosini L, Gelfo F (2022) Environmental enrichment effects on the brain-derived neurotrophic factor expression in healthy condition, alzheimer’s disease, and other neurodegenerative disorders. J Alzheimer’s Dis 85:975–992 34897089 10.3233/JAD-215193

[CR18] Galaj E, Barrera ED, Ranaldi R (2020) Therapeutic efficacy of environmental enrichment for substance use disorders. Pharmacol Biochem Behav 188:172829. 10.1016/j.pbb.2019.172829 31778722 10.1016/j.pbb.2019.172829 PMC6944776

[CR19] García-Rodríguez O, Ferrer-García M, Pericot-Valverde I et al (2011) Identifying specific cues and contexts related to smoking craving for the development of effective virtual environments. Cyberpsychol Behav Soc Netw 14:91–97. 10.1089/cyber.2010.0012 20575707 10.1089/cyber.2010.0012

[CR20] Janssen H, Ada L, Karayanidis F et al (2012) Translating the use of an enriched environment poststroke from bench to bedside: study design and protocol used to test the feasibility of environmental enrichment on stroke patients in rehabilitation. Int J Stroke 7:521–526. 10.1111/j.1747-4949.2011.00727.x 22264219 10.1111/j.1747-4949.2011.00727.x

[CR21] Kennedy RS, Lane NE, Berbaum KS, Lilienthal MG (1993) Simulator sickness questionnaire: an enhanced method for quantifying simulator sickness. Int J Aviat Psychol 3:203–220. 10.1207/s15327108ijap0303

[CR22] Kim H, Kim DJ, Kim S et al (2021) Effect of virtual reality on stress reduction and change of physiological parameters including heart rate variability in people with high stress: an open randomized crossover trial. Front Psychiatry 12. 10.3389/fpsyt.2021.614539 10.3389/fpsyt.2021.614539 PMC8384255 34447320

[CR23] Kumpulainen S, Esmaeilzadeh S, Pesola AJ (2024) Assessing the well-being benefits of VR nature experiences on group: heart rate variability insights from a cross-over study. J Environ Psychol 97:102366. 10.1016/j.jenvp.2024.102366

[CR24] Laviola G, Hannan AJ, Macrì S et al (2008) Effects of enriched environment on animal models of neurodegenerative diseases and psychiatric disorders. Neurobiol Dis 31:159–168. 10.1016/j.nbd.2008.05.001 18585920 10.1016/j.nbd.2008.05.001

[CR25] Liszio S, Masuch M (2019) Interactive immersive virtual environments cause relaxation and enhance resistance to acute stress. Annu Rev CyberTherapy Telemed 17:65–71

[CR26] Malińska M, Zużewicz K, Bugajska J, Grabowski A (2015) Heart rate variability (HRV) during virtual reality immersion. Int J Occup Saf Ergon 21:47–54. 10.1080/10803548.2015.1017964 26327262 10.1080/10803548.2015.1017964 PMC4536947

[CR27] McDonald MW, Hayward KS, Rosbergen ICM et al (2018) Is environmental enrichment ready for clinical application in human post-stroke rehabilitation? Front Behav Neurosci 12:1–16. 10.3389/fnbeh.2018.00135 30050416 10.3389/fnbeh.2018.00135 PMC6050361

[CR28] Nithianantharajah J, Hannan AJ (2006) Enriched environments, experience-dependent plasticity and disorders of the nervous system. Nat Rev Neurosci 7:697–709. 10.1038/nrn1970 16924259 10.1038/nrn1970

[CR29] Pang TY, Hannan AJ, Lawrence AJ (2019) Novel approaches to alcohol rehabilitation: modification of stress-responsive brain regions through environmental enrichment. Neuropharmacology 145:25–36. 10.1016/j.neuropharm.2018.02.021 29477298 10.1016/j.neuropharm.2018.02.021

[CR30] Paris MM, Carter BL, Traylor AC et al (2011) Cue reactivity in virtual reality: the role of context. Addict Behav 36:696–699. 10.1016/j.addbeh.2011.01.029 21349649 10.1016/j.addbeh.2011.01.029 PMC4104934

[CR31] Pericot-Valverde I, Germeroth LJ, Tiffany ST (2016) The use of virtual reality in the production of Cue-Specific craving for cigarettes: A Meta-Analysis. Nicotine Tob Res 18:538–546. 10.1093/ntr/ntv216 26453669 10.1093/ntr/ntv216

[CR32] Pratviel Y, Bouny P, Deschodt-Arsac V (2024) Immersion in a relaxing virtual reality environment is associated with similar effects on stress and anxiety as heart rate variability biofeedback. Front Virtual Real 5. 10.3389/frvir.2024.1358981

[CR33] Riva G, Mantovani, Fabrizia Capideville C, Preziosa A et al (2007) Affective interactions using virtual reality: the link between presence and emotions. Cyberpsychol Behav 10:45–56. 10.1089/cpb.2006.9993 17305448 10.1089/cpb.2006.9993

[CR34] Rockstroh C, Blum J, Göritz AS (2019) Virtual reality in the application of heart rate variability biofeedback. Int J Hum Comput Stud 130:209–220. 10.1016/j.ijhcs.2019.06.011

[CR35] Rodrigues J, Studer E, Streuber S et al (2020) Locomotion in virtual environments predicts cardiovascular responsiveness to subsequent stressful challenges. Nat Commun 11:1–11. 10.1038/s41467-020-19736-3 33214564 10.1038/s41467-020-19736-3 PMC7677550

[CR36] Rodrigues MJ, Postolache O, Cercas F (2021) Autonomic nervous system assessment based on HRV analysis during virtual reality serious games. In: Nguyen NT, Iliadis L, Maglogiannis I, Trawiński B (eds) Computational collective intelligence. ICCCI 2021. Springer International Publishing, Cham, pp 756–768. 10.1007/978-3-030-88081-1_57

[CR37] Rosenzweig MR, Bennett EL (1996) Psychobiology of plasticity: effects of training and experience on brain and behavior. Behav Brain Res 78:57–65. 10.1016/0166-4328(95)00216-2 8793038 10.1016/0166-4328(95)00216-2

[CR38] Rosenzweig MR, Bennett EL, Hebert M, Morimoto H (1978) Social grouping cannot account for cerebral effects of enriched environments. Brain Res 153:563–576. 10.1016/0006-8993(78)90340-2 698794 10.1016/0006-8993(78)90340-2

[CR39] Shaffer F, Ginsberg JP (2017) An overview of heart rate variability metrics and norms. Front Public Heal 5:1–17. 10.3389/fpubh.2017.00258 10.3389/fpubh.2017.00258 PMC5624990 29034226

[CR40] Solinas M, Thiriet N, Chauvet C, Jaber M (2010) Prevention and treatment of drug addiction by environmental enrichment. Prog Neurobiol 92:572–592. 10.1016/j.pneurobio.2010.08.002 20713127 10.1016/j.pneurobio.2010.08.002

[CR41] Spano G, Theodorou A, Reese G et al (2023) Virtual nature and psychological and Psychophysiological outcomes: A systematic review. J Environ Psychol 89:102044. 10.1016/j.jenvp.2023.102044

[CR42] van Dammen L, Finseth TT, McCurdy BH et al (2022) Evoking stress reactivity in virtual reality: A systematic review and meta-analysis. Neurosci Biobehav Rev 138:104709. 10.1016/j.neubiorev.2022.104709 35644278 10.1016/j.neubiorev.2022.104709

[CR43] van Praag H, Kempermann G, Gage FH (2000) Neural consequences of enviromental enrichment. Nat Rev Neurosci 1:191–198. 10.1038/35044558 11257907 10.1038/35044558

[CR44] Vecchiato G, Jelic A, Tieri G et al (2015) Neurophysiological correlates of embodiment and motivational factors during the perception of virtual architectural environments. Cogn Process 16:425–429. 10.1007/s10339-015-0725-6 26224275 10.1007/s10339-015-0725-6

[CR45] Wang D, Wang Y, Wang Y et al (2014) Impact of physical exercise on substance use disorders: A meta-analysis. PLoS ONE 9. 10.1371/journal.pone.0110728 10.1371/journal.pone.0110728 PMC4199732 25330437

[CR46] Woo CC, Leon M (2013) Environmental enrichment as an effective treatment for autism: a randomized controlled trial. Behav Neurosci 127:487–497 23688137 10.1037/a0033010

[CR47] Yin J, Yuan J, Arfaei N et al (2020) Effects of biophilic indoor environment on stress and anxiety recovery: A between-subjects experiment in virtual reality. Environ Int 136:105427. 10.1016/j.envint.2019.105427 31881421 10.1016/j.envint.2019.105427

[CR48] Yuan M, Guo YS, Han Y et al (2021) Effectiveness and mechanisms of enriched environment in post-stroke cognitive impairment. Behav Brain Res 410:113357. 10.1016/j.bbr.2021.113357 33989729 10.1016/j.bbr.2021.113357

