# Supplementary material for: Virtual reality environmental enrichment effects on heart rate variability in healthy volunteers
Source: Psychopharmacology (Berl). 2025 Jul 31;243(3):671–82. doi: 10.1007/s00213-025-06870-3 (PMC12979309; doi:10.1007/s00213-025-06870-3)
Supplement: Supplementary file 1 — Supplementary Material 1 [file 213_2025_6870_MOESM1_ESM.docx]

**Article title:** Virtual Reality Environmental Enrichment effects on Heart Rate Variability in healthy volunteers

**Journal:** Psychopharmacology

**Authors:** Giulia Benvegnù, Rudi Graffer, Federico Maria Lorusso, Sofia Ceccato, Erika Tedesco, Cristiano Chiamulera

**Corresponding author:** Giulia Benvegnù, Department of Diagnostics and Public Health, University of Verona, Verona, Italy, [giulia.benvegnu@univr.it](mailto:giulia.benvegnu@univr.it)

**Table S1 Correlations between HRV indexes and self-report measures**

|  | EE | | | CE | | |
| --- | --- | --- | --- | --- | --- | --- |
| Correlation | P-value | r | 95% CI | P-value | r | 95% CI |
| RMSSD ~ Arousal | 0.968 | 0.009 | -0.45, 0.47 | 0.699 | -0.095 | -0.54, 0.39 |
| RMSSD ~ Pleasantness | 0.321 | 0.24 | -0.25, 0.63 | 0.26 | 0.27 | -0.22, 0.65 |
| RMSSD ~ Immersion | 0.020 | 0.52 | 0.08, 0.79 | 0.959 | -0.013 | -0.48, 0.46 |
| RMSSD ~ Positive mood | 0.02 | 0.12 | -0.35, 0.56 | 0.565 | 0.14 | -0.35, 0.57 |
| RMSSD ~ Negative mood | 0.601 | -0.29 | -0.66, 0.19 | 0.18 | -0.32 | -0.68, 0.17 |
| HF ~ Arousal | 0.956 | 0.014 | -0.45, 0.48 | 0.84 | -0.05 | -0.50, 0.43 |
| HF ~ Pleasantness | 0.306 | 0.25 | -0.25, 0.64 | 0.2 | 0.31 | -0.18, 0.68 |
| HF ~ Immersion | 0.027 | 0.51 | 0.054, 0.79 | 0.449 | -0.18 | -0.60, 0.31 |
| HF ~ Positive mood | 0.524 | 0.16 | -0.33, 0.58 | 0.528 | 0.15 | -0.34, 0.58 |
| HF ~ Negative mood | 0.173 | -0.33 | -0.69, 0.16 | 0.126 | -0.36 | -0.71, 0.12 |
| LF/HF ~ Arousal | 0.891 | -0.034 | -0.49, 0.44 | 0.71 | -0.091 | -0.53, 0.39 |
| LF/HF ~ Pleasantness | 0.496 | -0.17 | -0.59, 0.32 | 0.121 | -0.37 | -0.71, 0.12 |
| LF/HF ~ Immersion | 0.431 | -0.19 | -0.60, 0.30 | 0.56 | 0.14 | -0.35, 0.57 |
| LF/HF ~ Positive mood | 0.67 | -0.1 | -0.54, 0.38 | 0.588 | -0.13 | -0.56, 0.35 |
| LF/HF ~ Negative mood | 0.203 | 0.31 | -0.19, 0.68 | 0.161 | 0.34 | -0.15, 0.69 |

**Legend.** EE: Enriched Environment, CE: Control Environment, RMSSD: Root Mean Square of Successive beat-to-beat interval Differences, HF: High Frequency; LF/HF: ratio of Low Frequency to High Frequency

**Table S2 Correlations between behavioral and self-report measures**

|  | Interaction | | | Deambulation | | | | | | |
| --- | --- | --- | --- | --- | --- | --- | --- | --- | --- | --- |
|  | EE | | | EE | | | CE | | |  |
| Correlation | P-value | r | 95% CI | P-value | r | 95% CI | P-value | r | 95% CI |  |
| Event ~ Arousal | 0.41 | -0.20 | -0.61, 0.29 | 0.26 | 0.26 | -0.22, 0.65 | 0.18 | 0.32 | -0.18, 0.68 |  |
| Event ~ Pleas. | 0.48 | -0.17 | -0.59, 0.32 | 0.024 | 0.52 | 0.067, 0.79 | 0.95 | -0.013 | -0.48, 0.46 |  |
| Event ~ Imm. | 0.26 | -0.27 | -0.65, 0.23 | 0.25 | 0.28 | -0.22, 0.66 | 0.339 | -0.23 | -0.63, 0.26 |  |
| Event ~ P. mood | 0.81 | -0.057 | -0.51, 0.42 | 0.08 | 0.40 | -0.07, 0.73 | 0,97 | -0.009 | -0.47, 0.46 |  |
| Event ~ N. mood | 0.72 | -0.087 | -0.53, 0.39 | 0.11 | -0.38 | -0.72, 0.11 | 0.72 | 0.086 | -0.40, 0.53 |  |
| Duration ~ Arousal | 0.31 | 0.25 | -0.25, 0.64 | 0.06 | -0.43 | -0.74, 0.04 | 0.99 | -0.0008 | -0.47, 0.46 |  |
| Duration ~ Pleas. | 0.19 | 0.31 | -0.18, 0.68 | 0.11 | -0.37 | -0.72, 0.11 | 0.95 | -0.0045 | -0.47, 0.46 |  |
| Duration ~ Imm. | 0.044 | 0.47 | 0.0002, 0.77 | 0.58 | -0.13 | -0.56, 0.35 | 0.47 | 0.18 | -0.31, 0.59 |  |
| Duration ~ P. mood | 0.17 | 0.33 | -0.16, 0.69 | 0.52 | -0.15 | -0.58, 0.34 | 0.39 | 0.21 | -0.28, 0.61 |  |
| Duration ~ N. mood | 0.19 | -0.31 | -0.68, 0.18 | 0.85 | 0.04 | -0.43, 0.50 | 0.71 | -0.08 | -0.53, 0.39 |  |
| E % t ~ Arousal | 0.67 | 0.10 | 0.38, 0.54 | 0.91 | -0.02 | -0.48, 0.44 | 0.12 | 0.37 | -0.10, 0.70 |  |
| E % t ~ Pleas. | 0.22 | 0.29 | 0.20, 0.67 | 0.37 | 0.22 | 0.28, 0.62 | 0.62 | 0.12 | -0.36, 0.56 |  |
| E % t ~ Imm. | 0.25 | 0.27 | 0.22, 0.66 | 0.788 | 0.06 | -0.41, 0.52 | 0.635 | 0.12 | -0.37, 0.55 |  |
| E % t ~ P. mood | 0.14 | 0.35 | -0.14, 0.70 | 0.71 | 0.08 | -0.39, 0.53 | 0,28 | 0.26 | -0.24, 0.65 |  |
| E % t ~ N. mood | 0.73 | -0.42 | -0.74, 0.05 | 0.29 | -0.25 | -0.64, 0.24 | 0,92 | -0.023 | -0.48, 0.45 |  |

**Legend.** EE: Enriched Environment, CE: Control Environment, Event: number of events, Pleas.: pleasantness, Imm.: immersion, P. mood: positive mood, N. mood: negative mood, Duration: average duration, E % t: event % time

**Supplementary results: activity in virtual environments divided by groups**

The Wilcoxon signed rank tests performed on the different walking indices divided by groups revealed that the average duration (z = 2.584, p = 0.010, r = 0.81) and the percentage of session time spent walking (z = 2.755, p = 0.006, r = 0.87) were significantly higher in the CE scenario than in the EE scenario only in group B (CE, EE). Group A (EE, CE) showed no significant differences in the two indices.
